# Terahertz Reflectometry Imaging of Carbon Nanomaterials for Biological Application

**DOI:** 10.35248/2157-7439.19.10.535

**Published:** 2019-08-26

**Authors:** William Ghann, Hyeonggon Kang, Aunik K Rahman, Anis Rahman, Meser M Ali, Jamal Uddin

**Affiliations:** 1 Department of Natural Sciences, Center for Nanotechnology, Coppin State University, 2500 W. North Ave, Baltimore, MD, USA;; 2 Applied Research & Photonics Inc., 470 Friendship Road, Suite 10, Harrisburg, PA 17111, USA;; 3 Cellular and Molecular Imaging Laboratory, Department of Neurosurgery, Henry Ford Hospital, Detroit, MI, USA

**Keywords:** Nanomaterials, Imaging, Nanotubes

## Abstract

The multiwalled carbon nanotubes has a myriad of applications due to its unique electrical and mechanical properties. The biomedical application of multiwalled carbon nanotubes that have been reported include drug delivery, medical imaging, gene delivery, tissue regeneration, and diagnostics. Proper characterization is required to enhance the potential application of the multiwalled carbon nanotubes. Terahertz technology is a relatively unfamiliar spectrometric technique that show promise in efficiently characterizing multiwalled carbon nanotubes. In this paper, terahertz imaging was used to characterize multiwalled carbon nanotube in comparison with other characterization techniques, including transmission electron microscopy and field emission scanning electron microscopy. The average diameter of the carbon nanotubes from the reconstructed terahertz images was 48.54 nm, while the average length of a fiber was found to be approximately 1.2 μm. The multiwalled carbon nanotubes were additionally characterized by FTIR, Raman spectroscopy, and Energy-dispersive X-ray spectroscopy.

## INTRODUCTION

Carbon nanotubes are a hollow, cylindrically-shaped, nanostructured allotrope of carbon. There are two types of carbon nanotubes based on their structure: 1) single-walled carbon nanotube (SWCNTs), which consist of a sheet of graphene that are rolled up into a cylinder, and 2) multiwalled carbon nanotubes (MWCNTs), which consist of more than two graphene sheets rolled together to form a cylinder. Carbon nanotubes have unique mechanical and electrical properties that lend themselves to extraordinary applications in electronics, optics, and thermal activities. They have also been used in medicine in the thermal ablation of cancerous tissues, among others [1–7]. One other important application is their use in the removal of different kinds of contaminants in water bodies [8]. Carbon nanotubes (CNTs) were discovered and first described by Iijima et al in 1991 [9]. Since this discovery, several techniques and instrumentation have been employed to characterize them. Notable among these characterizing tools are X-ray photoelectron spectroscopy (XPS), Transmission Electronic Microscopy(TEM), Fourier Transform Infrared Spectroscopy (FTIR), and Raman spectroscopy [10–18].

Terahertz is a very important but rarely explored portion of the electromagnetic spectrum. It lies between the microwave and infrared region and has been shown to interact with a wide range of materials [19]. For this reason, terahertz radiation has found use in spectroscopy and imaging of very important materials [20]. Terahertz (THz) technology applications include security screening, medicine, bioengineering, pharmacy, astronomy, environmental monitoring, dentistry, and communications [20,21]. Imaging various materials with terahertz has it own challenges as resolution of the images is adversely affected by the long wavelength of the terahertz radiation. The resolution is thus usually in the order of a couple of hundred microns. Terahertz has attracted a lot of attention in recent years due to improvements in the developement of sources and devices for detection of the radiation [22–24].

Carbon nanotubes have previously been used as component of terahertz system [25–30]. However, in this paper, terahertz is used to characterize multiwalled carbon nanotubes through terahertz reflectometry Imaging. Thus this study is the first of its kind to shed light on the characterization of multiwalled carbon nanotubes using terahertz reconstructive imaging. The multiwalled carbon nanotubes is analyzed using the terahertz radiation generated by via a mechanism called dendrimer dipole excitation (DDE) in which an electro-optic dendrimer is excited by a pump laser to generate a continuous wave (CW) terahertz radiation.

## EXPERIMENTAL SECTION

The morphology and elemental composition of the multiwalled carbon nanotubes was analyzed using field emission scanning electron microscopy equipped with an energy-dispersive X-ray spectroscopy analyzer (Model FESEM: JSM-7100FA JEOL USA, Inc.). Raman Measurements were performed on a model DXR Smart Raman Spectrometer (Thermo Fisher Scientific Co., Ltd., USA). FTIR spectra were obtained with a Thermo Nicolet iS50 FTIR (Thermo Fisher Scientific Co., Ltd., USA). Transmission Electron Microscopy (TEM) images were acquired on JEM-1400 PLUS (JEOL USA, Peabody, Massachusetts, USA). The images were viewed using Digital Micrograph software from GATAN (GATAN Inc., Pleasanton, CA, USA).

**Figure 1:**
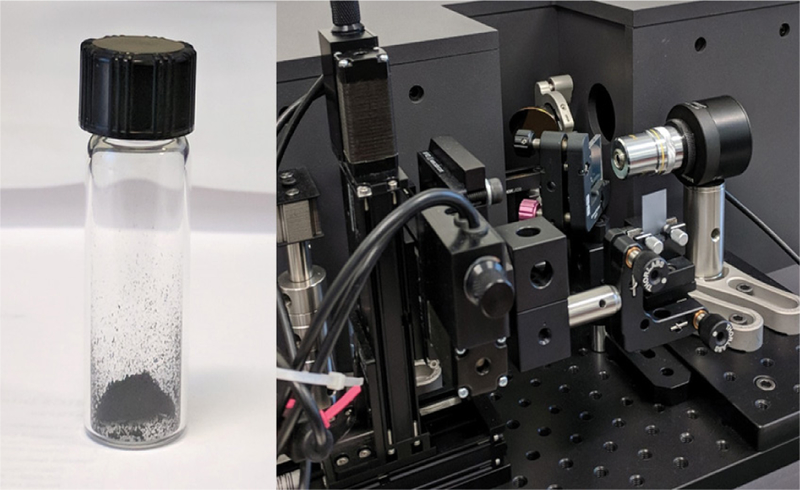
MWCNTs used in the study and the terahertz instrument used to characterize the sample. (left: MWCNTs in glass vial; right: Terahertz instrument equipped with nanoscanner for imaging).

The CNT sample used in the study, purchased from Sigma Aldrich, TCI AMERICA, Portland, USA, is displayed in Figure 1. To carry out some of the measurements, the multiwalled carbon nanotubes were dispersed in water (Figure 1) also shows the terahertz instrument used in carrying out the measurement.

The process of terahertz generation involves an electro-optic dendrimer which is excited by a pump laser, where continuous wave (CW) terahertz radiation is generated via a mechanism called dendrimer dipole excitation (DDE). This DDE source generates stable terahertz radiation over a range of ~0.1 THz to ~30 THz at room temperature. Measurements are carried out either in the transmission mode or reflectance mode. The navigation between the two modes is facilitated by the nanoscanner component of the instrument. The measurements are carried out in a reflection mode for 3D imaging. The 3D motion of the sample holder is facilitated by the nanoscanner enabling the interrogation of the reflectance across all the three axes of the sample. The nanoscanner also positions a sample in the path of the beam for transmission measurements and also facilitates 3D scanning in the reflectance mode.

During 3D terahertz scanning reflectometry imaging measurements, the terahertz beam of TeraSpectra first hits the off-axis parabolic reflector and is focused on the sample at a 90-degree angle. The reflected beam from the sample is directed to the detection system via the beam splitter. The surface plots and 3D images of the multiwalled carbon nanotubes were generated by means of Voxler® 4 visualization software from Golden Software Inc.

## RESULTS AND DISCUSSION

The multiwalled carbon nanotubes were characterized using the terahertz reconstructive imaging. To confirm the authenticity of the MWCNTs, the nanomaterial was also analyzed with Field Emission Scanning Electron Microscopy, Energy-dispersive X-ray spectroscopy, Fourier Transform Infrared, Raman, and UV-visible Spectroscopy. Multiwalled carbon nanotubes have been characterized previously using terahertz technology. Multiwalled carbon nanotubes have also been used as component of terahertz machines used to generate and detect terahertz radiation. In this paper, terahertz technology is used to image the carbon nanotubes and to study the dimensions of the carbon nanotube.

**Figure 2:**
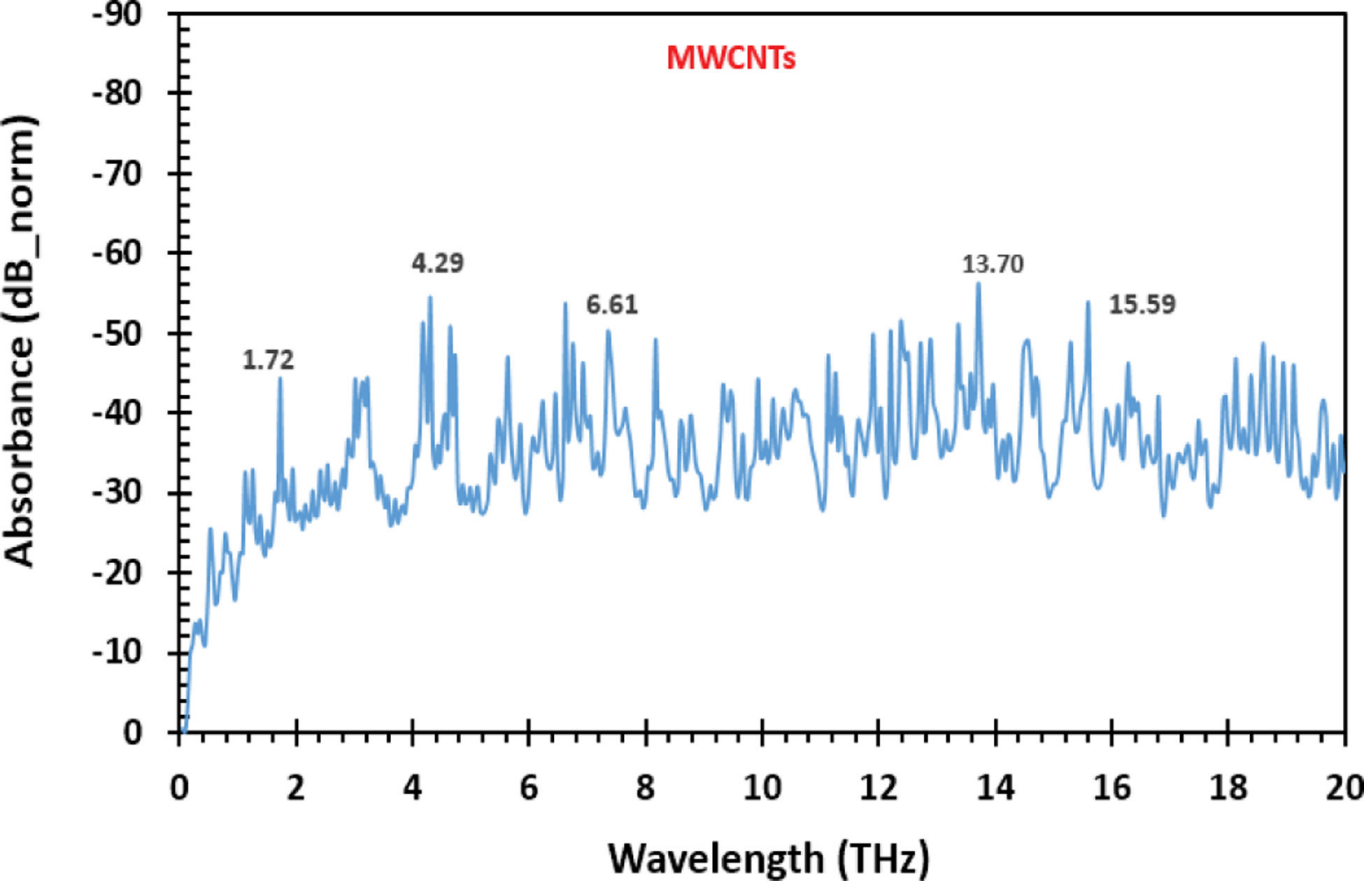
Fourier transforms broadband terahertz absorbance spectra of MWCNTs.

The result of the spectra analysis is displayed in Figure 2. The data is collected as the time domain signal, and then Fourier transformed to form the spectra data as displayed in Figure 2. Prominent peaks seen in the spectrum include 1.72 THz, 4.29 THz, 6.61 THz, 13.70 THz, and 15.59 THz. The THz-TDS spectrometer generates data first in the time domain to which Fourier transform algorithms is applied to obtain a frequency spectrum. The data was collected within the range of 0 and 50 THz, nonetheless, to increase legibility, only spectra up to 5 THz are displayed.

### Fourier transformed infrared studies

**Figure 3:**
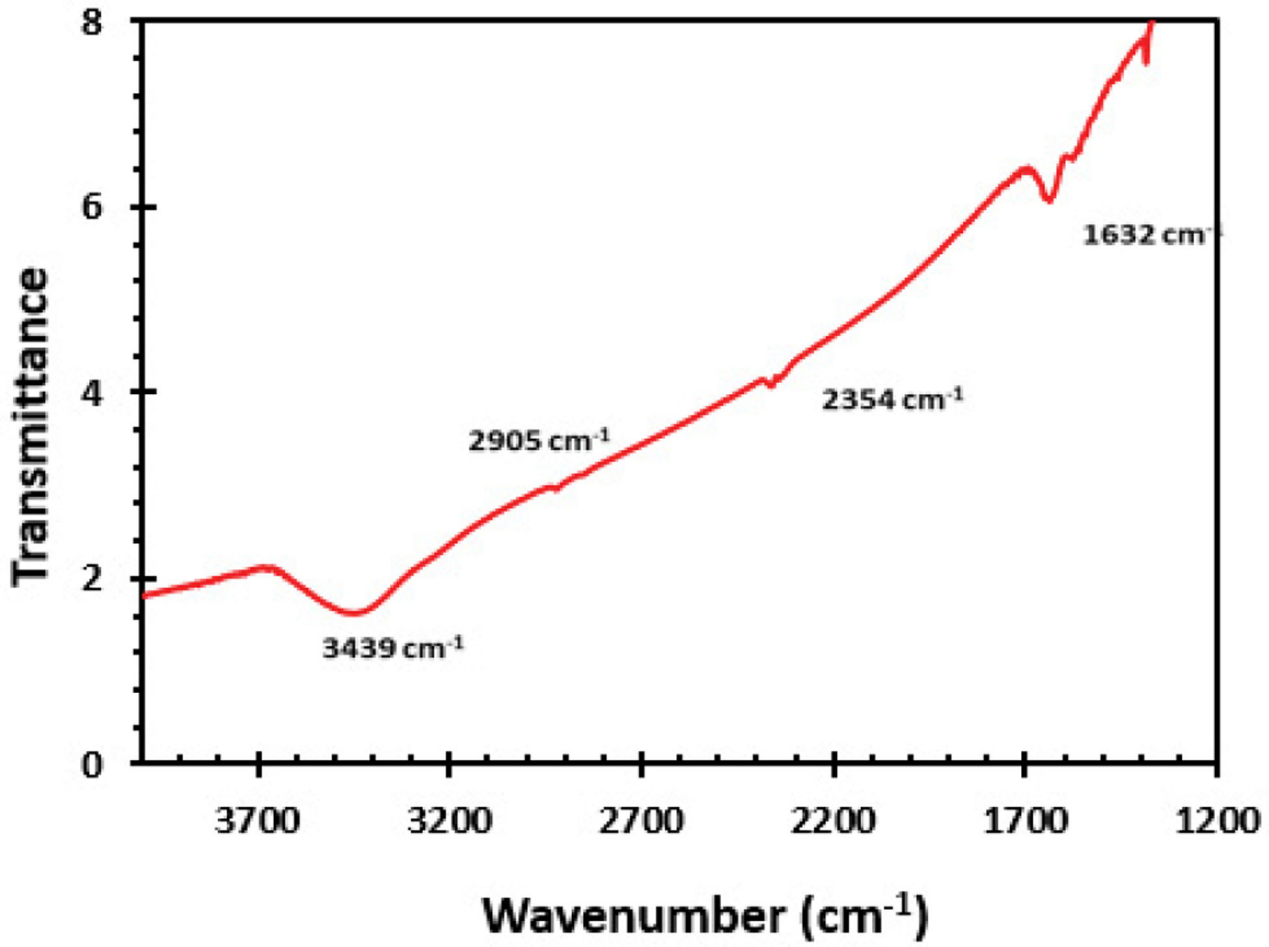
FTIR Spectra of multiwalled carbon nanotubes.

Figure 3 demonstrates the FTIR spectra of MWCNT. The FTIR measurement was carried out in the range of 500–4000 cm^−1^. The spectra show peaks corresponding to various bonds in the samples [18]. The FTIR spectrum of MWCNT displays bands at 3439 cm^−1^, 2905 cm^−1^, 2354 cm^−1^, and 1632 cm^−1^. KBr pellets of the multiwalled were prepared for FTIR analysis. The peak at 1632 cm^−1^ is assigned to C=C stretching of CNT structure. The band at 3439 cm^−1^ could be attributed to the O-H vibration associated with amorphous carbon. The peak at 1383 cm^−1^ likely corresponds to the C-O stretching vibration that could be associated with defects in multiwalled carbon nanotubes [13,19].

### Raman studies of the MWCNTs

**Figure 4:**
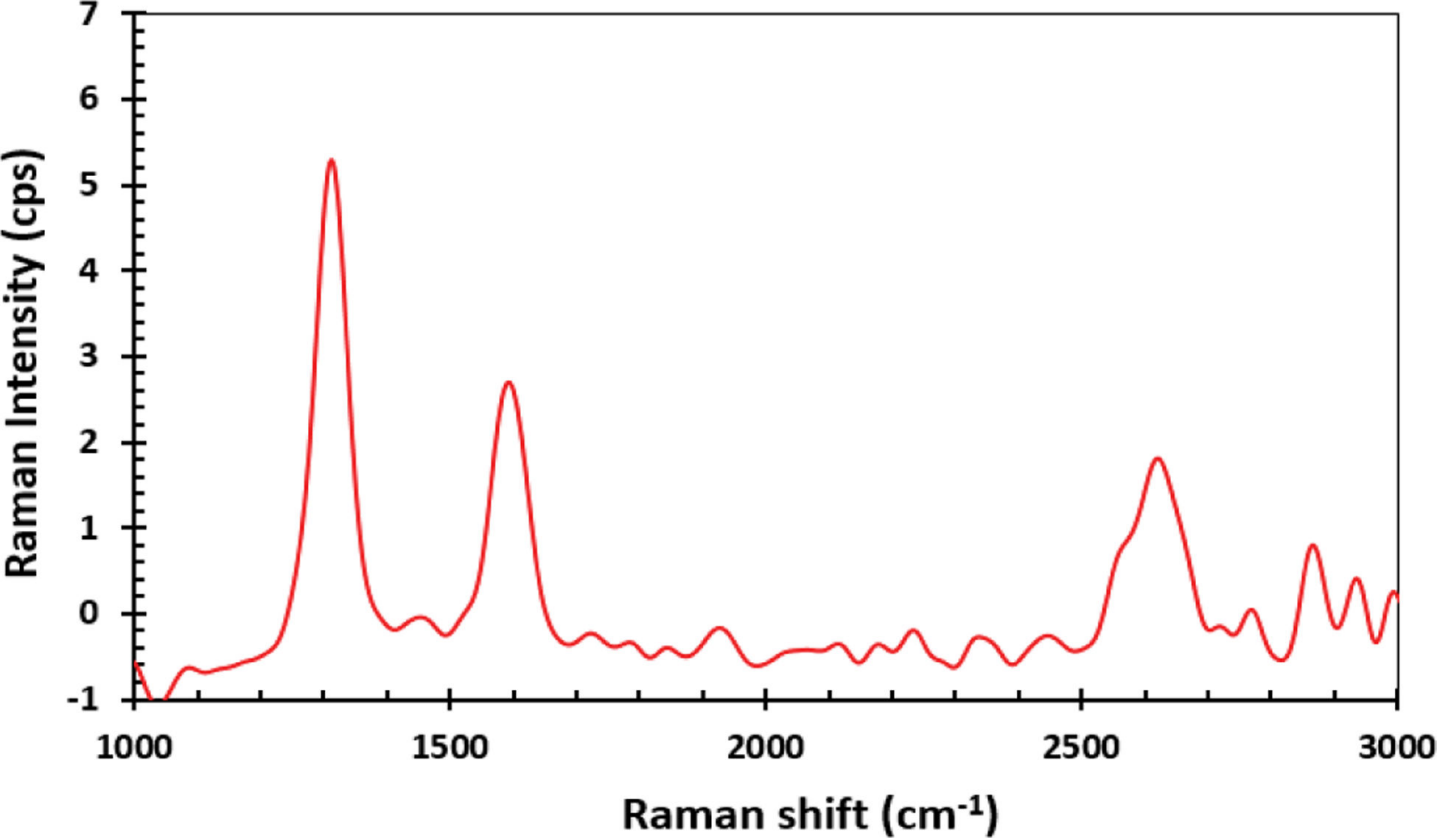
Raman Spectra of the MWCNTs.

The sample was further characterized by Raman spectroscopy. The spectra of the measurements is displayed in Figure 4. Three main peaks were observed in the spectra which is consistent with the expected Raman signals of the MWCNTs. The peaks of interest are 1314 cm^−1^, 1593 cm^−1^, and 2619 cm^−1^. They are consistent with other published data on Multiwalled carbon nanotubes. The band at 1314 cm^−1^, also known as the D band, corresponds to the degree of structural disorder of the nanotubes. The band at 1593 cm^−1^ is the G band, which corresponds to the degree of nanotubes graphitization, whereas the band at 2619 cm^−1^, also known as the 2D band, generally correspond to stresses. The shapes and ratio of the D and B modes confirms the presence of multiwalled carbon nanotubes [20].

### Energy-dispersive x-ray spectroscopy (EDS) studies

**Figure 5:**
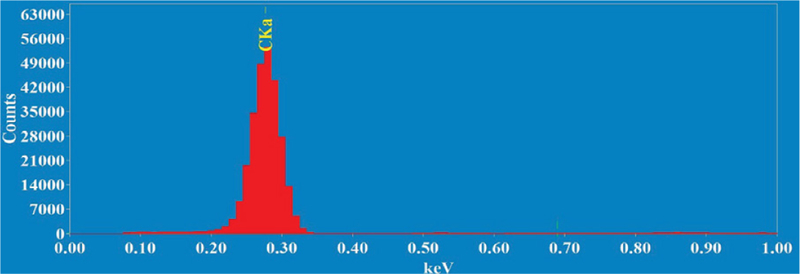
Energy-dispersive X-ray spectroscopy of the MWCNTs.

Energy-dispersive X-ray Spectroscopy (EDS) studies were carried out to confirm the elements present in the sample and to identify any impurity that might be present in the sample. The EDS spectra of the sample is displayed in Figure 5. The only element present, consistent with the composition of the samples, was carbon.

### Field emission scanning electron microscopy (FESEM) imaging

**Figure 6:**
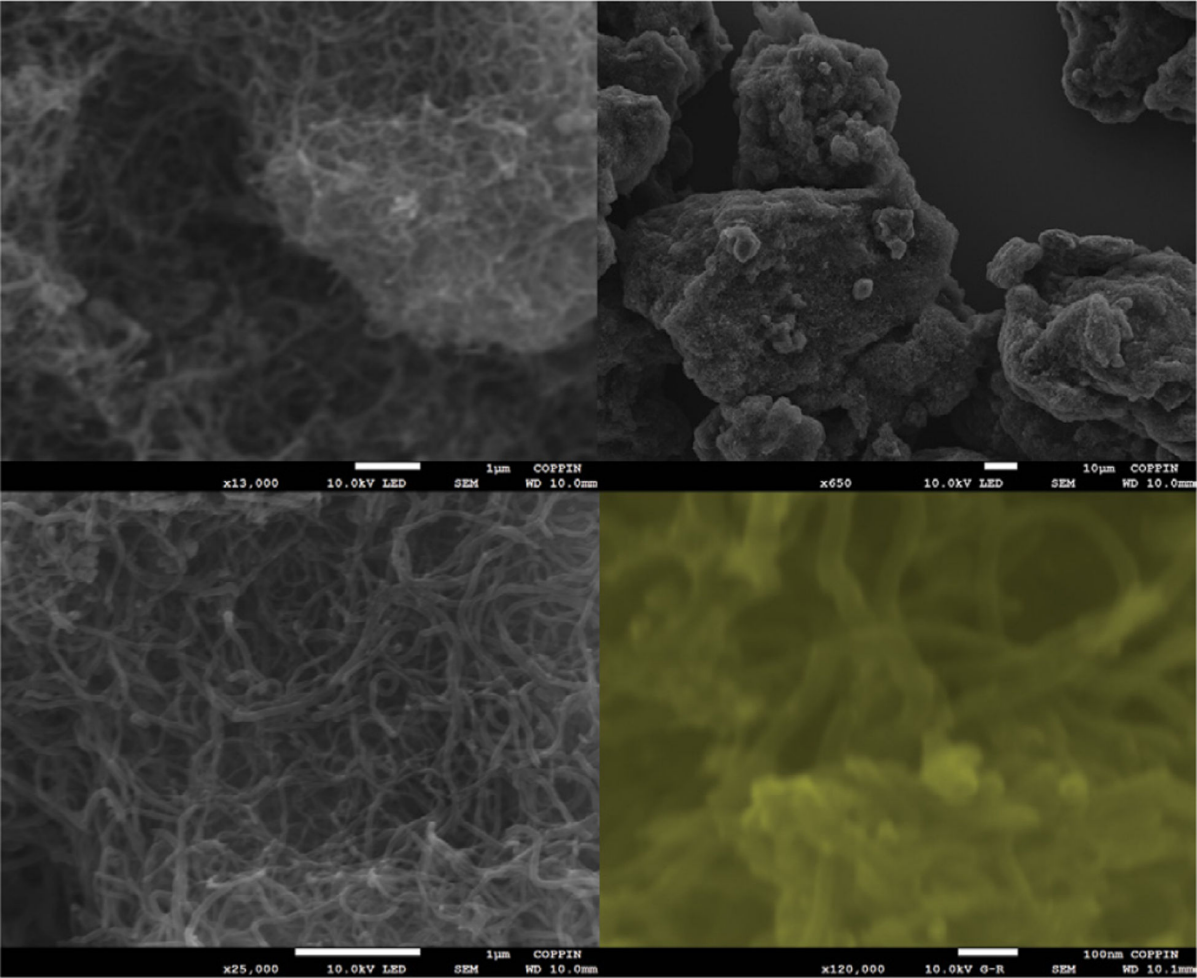
Field Emission Scanning Electron Microscopy (FESEM) images of the MWCNTs taken at different magnification. Length ~1.2 μm and diameter ~47 nm.

The sample was characterized via Field Emission Scanning Electron Microscopy (FESEM) imaging. The measurement was carried out to determine the length and diameter of the multiwalled carbon nanotubes, and to observe the porosity and morphology of the sample. FESEM images of the samples at different magnifications are displayed in Figure 6. The average length of the MWCNTs was determined to be 1.2 μm and the diameter average out at 57 nm.

### Transmission electron microscopy imaging

**Figure 7:**
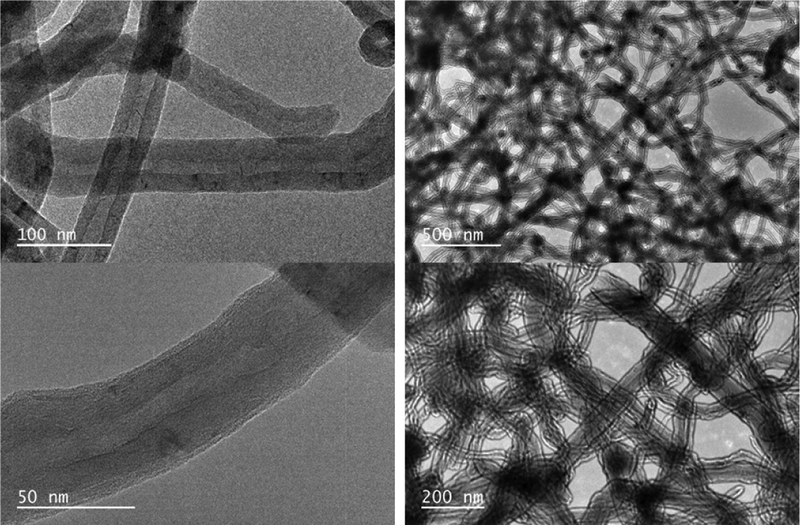
TEM image of the multiwalled carbon nanotubes taken at different magnification.

The MWCNTs were further characterized using transmission electron microscopy. This characterization was carried out to confirm the diameter and length of the nanotubes. Figure 7 shows the transmission electron microscopy image of the multiwalled carbon nanotubes. The MWCNTs as seen on the TEM image consist of many fibers tangled together. Each tube consist of two dark lines at the edges and a space between the two. There are also variation in the length of the carbon nanotubes. Since the fibers are twisted together, it is difficult to determine the exact the length of the fibers. However, the average length was about 1.2 μm whereas the average diameter was 45 nm.

### Terahertz reconstructive imaging of MWCNTs

Reconstructive imaging provides a substitute for the CCD based imaging. Though CCDs, such as digital microscope and cameras, normally have good resolution, they also have limitations for achieving these high level of resolution. Also, it is strictly a surface imaging device. TEM offers high resolution imaging. However, it is strictly a destructive technique with laborious sample preparation requirements, and only for small geometries. Other techniques, such as focused ion beam, XRD, etc., are also destructive techniques. Reconstructive imaging offers an important opportunity to define the pixel size by a hardware and software combination. A number researchers have used terahertz spectroscopy to characterize single-walled carbon nanotubes and have reported unique spectra features for the carbon nanotubes [20–24]. In this novel work, multiwalled carbon nanotubes were characterized using the terahertz reconstructive imaging.

**Figure 8:**
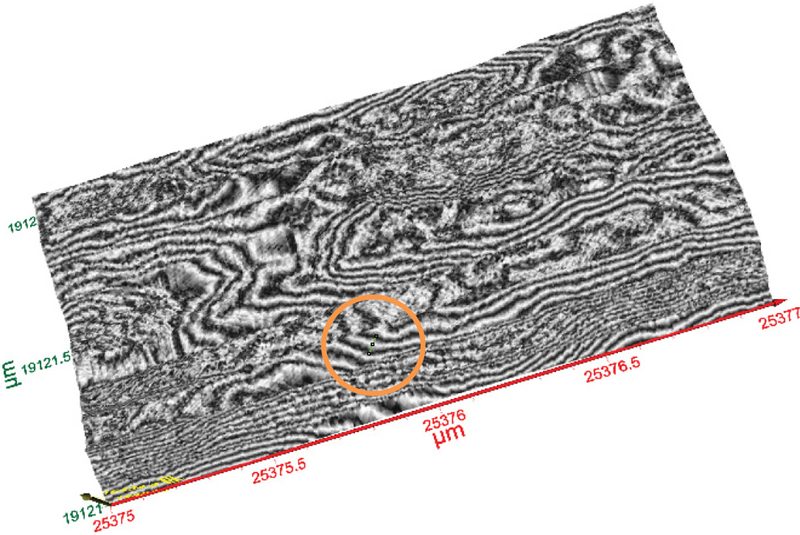
A representative 3D image of a section of MWCNTs. A single CNT’s width is shown by the line in the circle.

**Figure 9:**
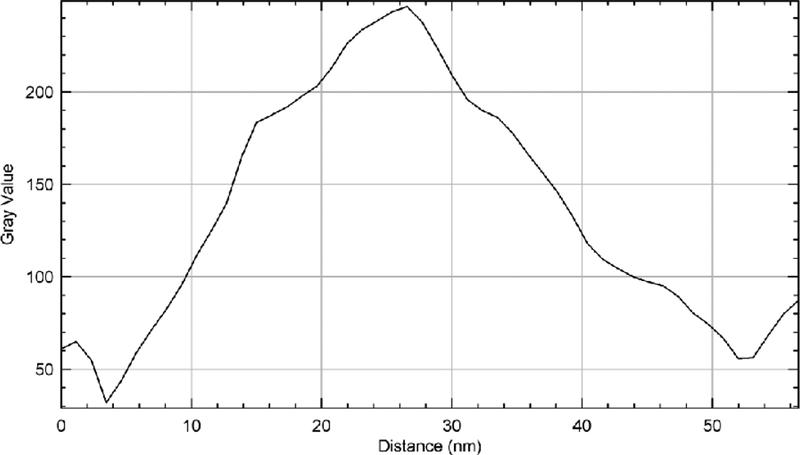
Size analysis of the image of MWCNTs. Average diameter of a single CNT=48.54 nm (edge to edge), see the circle in Figure 8.

**Figure 10:**
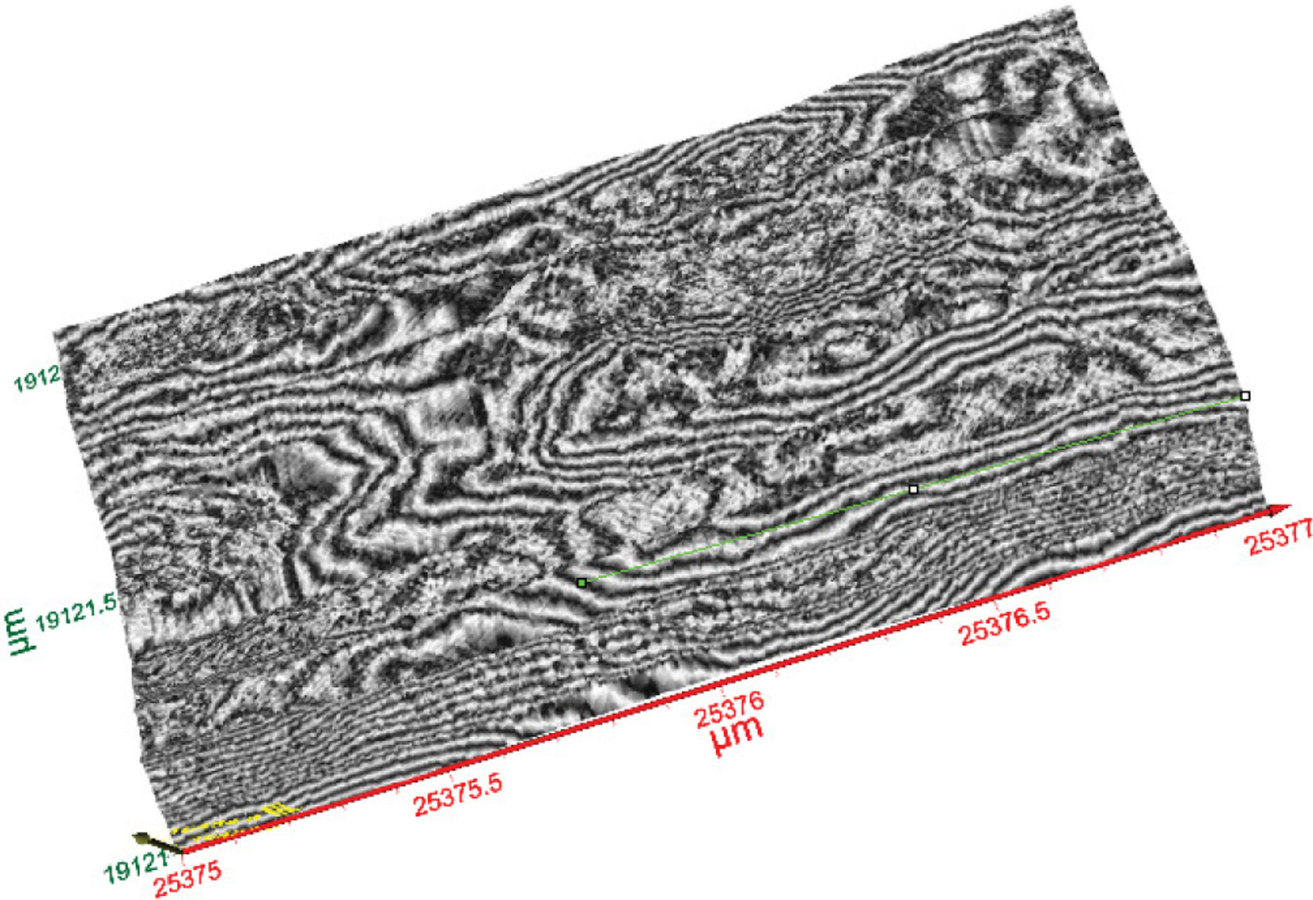
The same image as in Figure 8 is used for length estimate of a single MWCNT (green line).

**Figure 11:**
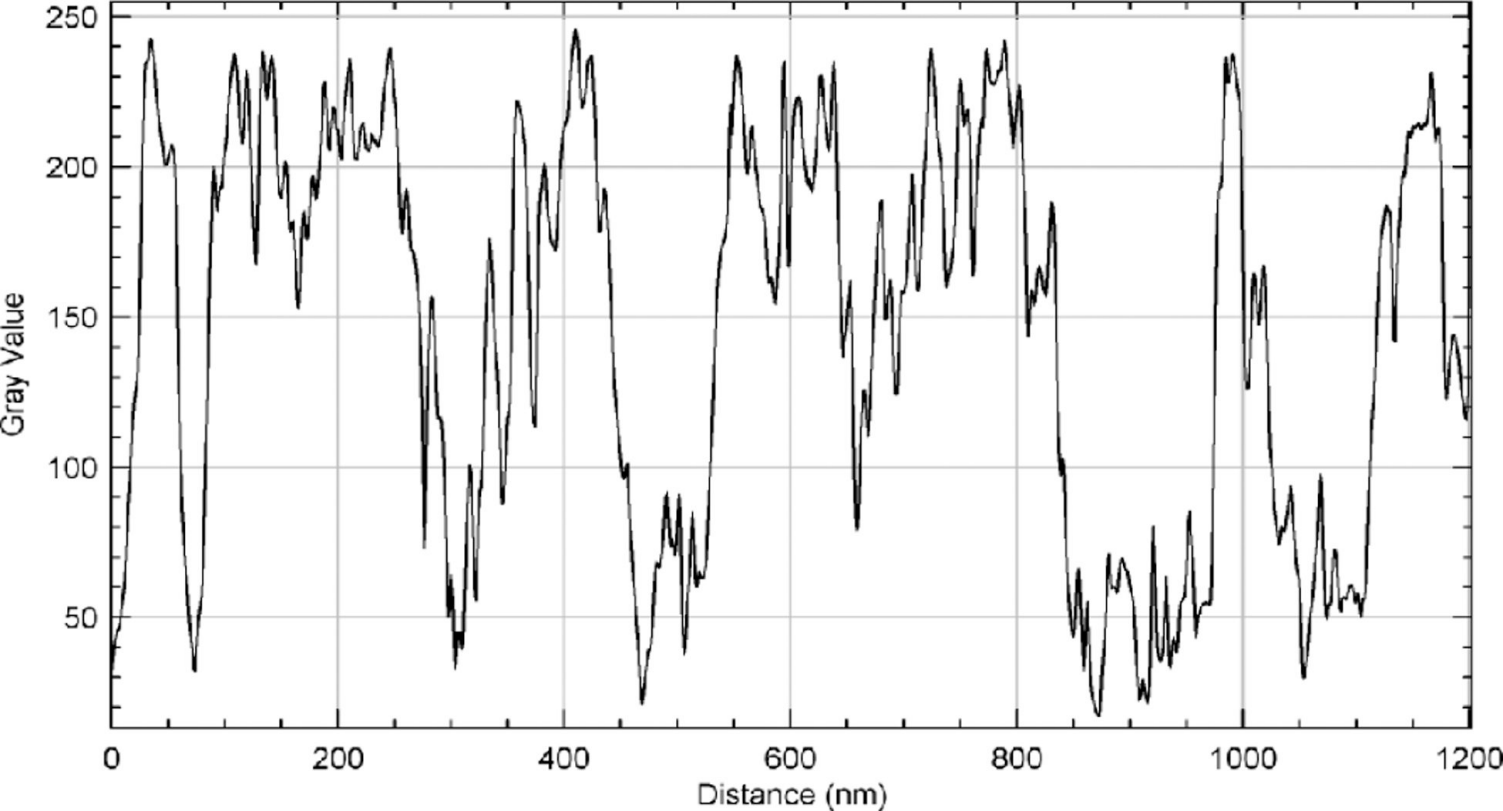
Length determination of MWCNT from Figure 8. Length of a single MWCNT=~1200 nm.

**Figure 12:**
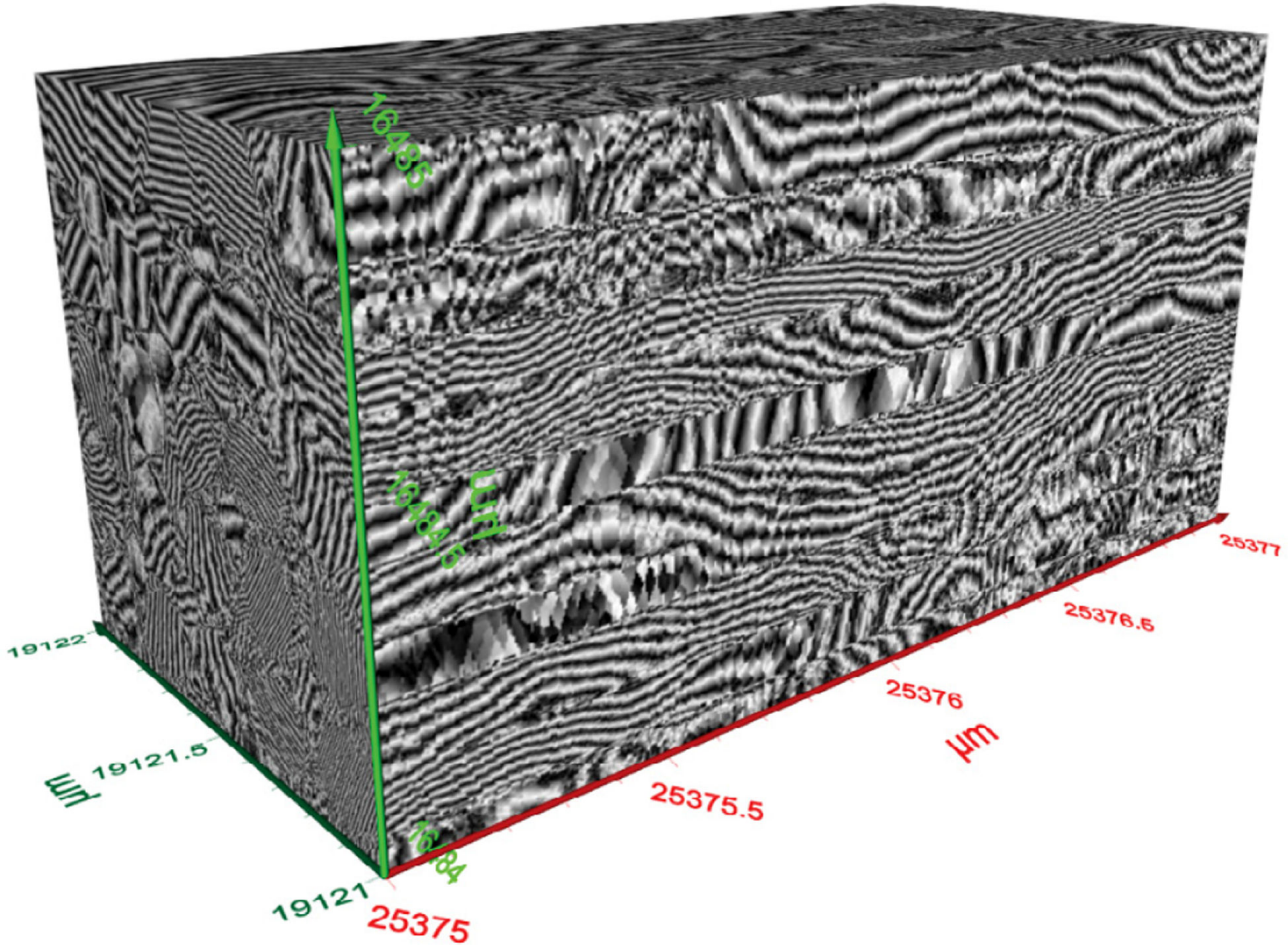
3D image of a section of MWCNT. It reveals the 3D organization of the annealed CNTs on the substrate.

Figure 8 shows a representative 3D image of a section of MWCNTs showing various strands of the MWCNTs. The portion imaged was a 2 μm by 1 μm section of the carbon nanotubes. A single CNT’s width is shown by the line in the orange circle (Figure 8). The size analysis of the image of MWCNTs displayed in Figure 10 which is demonstrated in Figure 9. Based on the analysis, the average diameter of a single CNT was determined to be 48.54 nm. As displayed within the circle shown in Figure 8, the diameter was determined from one edge to another edge of the MWCNT fiber. In addition to the diameter, the length of each MWCNT was also probed. Figure 8 shows the section of the MWCNT imaged to determine the length of a fiber of MWCNTs that was investigated. The actual size analysis for the computation of the length of a strand of the MWCNTs is shown in Figure 11. The 3D organization of the annealed 3D image of is displayed in Figure 12.

## CONCLUSION

In this study, terahertz technology was used to characterize multiwalled carbon nanotubes. The diameter and the length of the carbon nanotubes were determined through terahertz reconstructive imaging. The diameter of the carbon nanotubes was measured to be 48.54 nm and length determined to be 1.2 μm. The multiwalled carbon nanotubes were also characterized with Field Emission Scanning Electron Microscope and Transmission Electron Microscope. The dimensions obtained with the FESEM and TEM were similar to that of the terahertz reconstructive imaging. Finally, the carbon nanotubes were also characterized with FTIR, Raman, UV-Vis, and EDS to confirm the elemental composition of the sample. This work to the best our knowledge is the first to show the use of terahertz reconstructive imaging for the size charaterization of multiwalled carbn nanotubes.
